# Genetic analysis of a Piezo-like protein suppressing systemic movement of plant viruses in *Arabidopsis thaliana*

**DOI:** 10.1038/s41598-019-39436-3

**Published:** 2019-02-28

**Authors:** Zhen Zhang, Xin Tong, Song-Yu Liu, Long-Xiang Chai, Fei-Fan Zhu, Xiao-Peng Zhang, Jing-Ze Zou, Xian-Bing Wang

**Affiliations:** State Key Laboratory of Agro-Biotechnology, College of Biological Sciences, China Agricultural University, Beijing, 100193 China

## Abstract

As obligate intracellular phytopathogens, plant viruses must take advantage of hosts plasmodesmata and phloem vasculature for their local and long-distance transports to establish systemic infection in plants. In contrast to well-studied virus local transports, molecular mechanisms and related host genes governing virus systemic trafficking are far from being understood. Here, we performed a forward genetic screening to identify *Arabidopsis thaliana* mutants with enhanced susceptibility to a 2b-deleted mutant of cucumber mosaic virus (CMV-2aT∆2b). We found that an uncharacterized Piezo protein (AtPiezo), an ortholog of animal Piezo proteins with mechanosensitive (MS) cation channel activities, was required for inhibiting systemic infection of CMV-2aT∆2b and turnip mosaic virus tagged a green fluorescent protein (GFP) (TuMV-GFP). *AtPiezo* is induced by virus infection, especially in the petioles of rosette leaves. Thus, we for the first time demonstrate the biological function of Piezo proteins in plants, which might represent a common antiviral strategy because many monocot and dicot plant species have a single Piezo ortholog.

## Introduction

Plant viruses are obligate parasites being largely dependent on their host for translation, replication, encapsidation, movement, and long-distance infection. Phloem transport provides the fastest way for plant viruses to spread throughout their host plants. During long-distance transport, there are three main steps including viral entry into phloem tissues, movement within sieve elements, and exit into cells of the sink tissues (review in references)^1,2^. Thus, virus resistance of host plants can be achieved by blocking these steps. For instance, Ueki and Citovsky have showed that a cadmium ion-induced glycine-rich protein inhibited systemic infections of tobamoviruses by promoting callose deposition in the plant cell walls^3,4^. In addition to cadmium ion, mechanosensitive (MS) ion channels-mediated Ca^2+^ influx into sieve elements is usually induced and confers a quick defense response in plants undergoing biotic challenges^5^.

Cucumber mosaic virus (CMV) has a tripartite genome consisting of RNA1, RNA2 and RNA3, which encoded 1a protein possessing the helicase and methylase domain, an RNA-dependent RNA polymerase, and the viral movement protein (MP), respectively. In addition, RNA4, a subgenomic RNA produced from RNA3, encodes viral coat protein (CP), while RNA4A, a subgenomic RNA derived from RNA2, encodes the viral suppressor of RNA silencing (VSR), namely the 2b protein^6–8^. *A*. *thaliana* wild-type plants and mutant plants loss of antiviral genes usually exhibit similar response to wild-type CMV due to strong VSR activities of 2b^9^. Thus, 2b-deficient mutant viruses were recently employed to facilitate screening some antiviral components through forward genetics^10–14^. CMV-2aTΔ2b, a CMV mutant with a 295-nt deletion in its 2b coding region and the overlapping C-terminus of CMV 2a protein, induces secondary siRNA amplification mediated by host RNA-dependent RNA Polymerase 1 (RDR1) and RDR6^12,13^.

Turnip mosaic virus (TuMV) genomic RNA is an approximately 10-kb single stranded RNA encoding a polyprotein, which is cleaved to produce 11 mature proteins^15^. NIb functions as a polymerase and 6K2 is related to formation of replication vesicles^16^. P3N-PIPO together with cylindrical inclusion protein (CI), CP, viral genome linked protein (VPg) and helper component-protease (HC-Pro) are involved in cell-to-cell movement and systemic spread^16–18^. The recombinant TuMV-GFP containing a GFP insertion between P1 and HC-Pro is usually used to elucidate the mechanisms and host factors during virus infection^15,18,19^.

The mouse Piezo1 protein functions as a Ca^2+^ permeable channel, regulating the volume of blood cell, integration of vascular architecture, differentiation of neural stem cells, and stretch induced cell proliferation^20–23^. Most plant species harbor a single Piezo protein^24^, which share high similarities with animal piezo proteins^24–28^. However, biological functions of plant Piezo orthologs are still poorly understood. In this study, the M2 population of ethyl methanesulfonate (EMS)-mutagenized Col-0 plants were used to screen enhanced susceptibility to CMV-2aTΔ2b (esc) mutants. We isolated a novel mutant, named *esc1*, which is more sensitive to the CMV-2aTΔ2b infection and TuMV-GFP than wild-type Col-0 plants. *ESC1* encodes a Piezo-like protein, which shows high similarity with mammalian Piezo proteins. Further analysis revealed that ESC1 limited the systemic movement of plant viruses.

## Results

### Identification and characterization of *esc1* mutant from forward genetic screening

**Figure 1:**
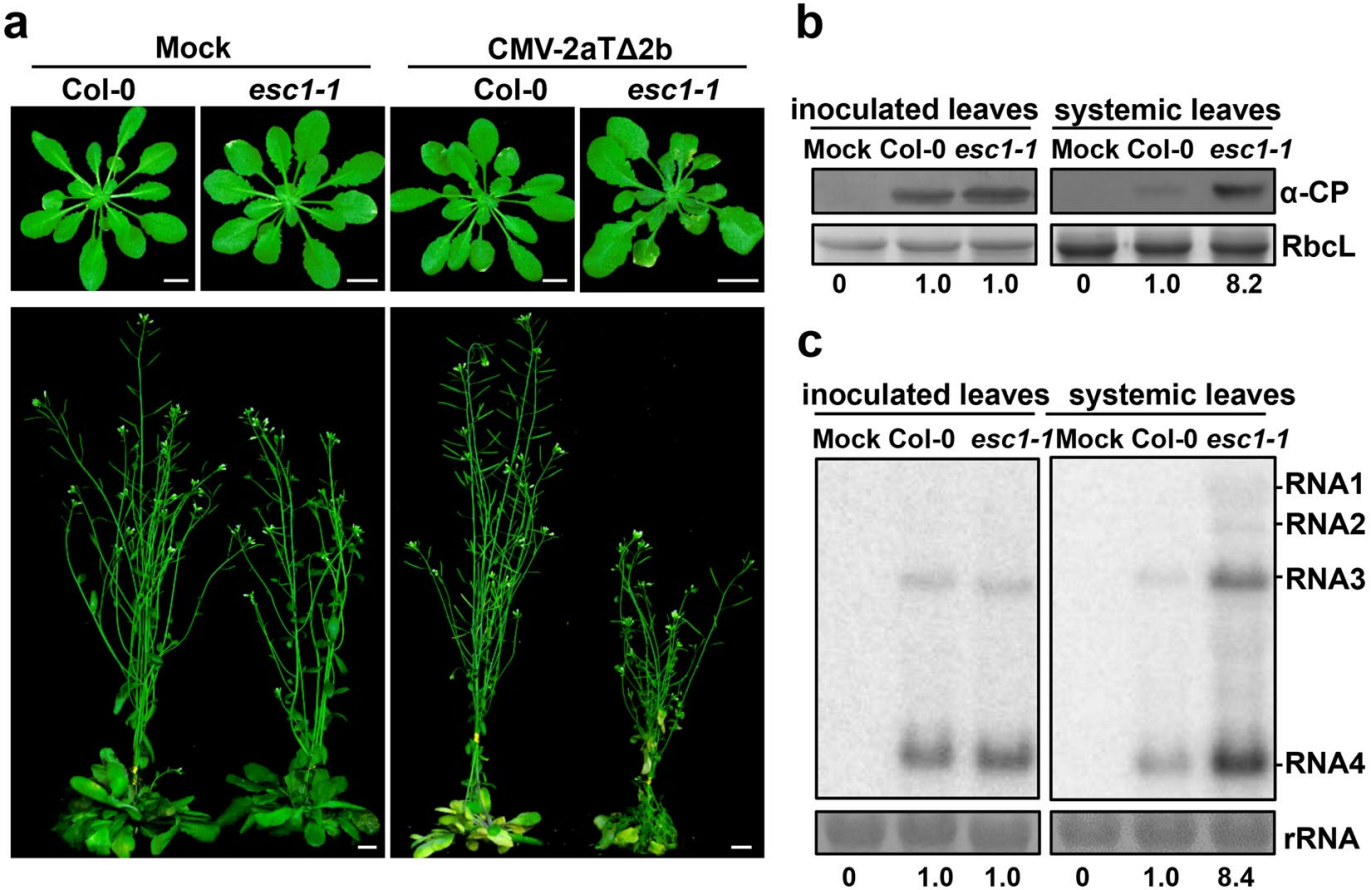
The *A*. *thaliana* mutant *esc1-1* showed enhanced susceptibility to CMV-2aTΔ2b. (**a**) Pathogenetic responses of Col-0 and *esc1-1* to the CMV-2aTΔ2b infection or mock treatment. Seedlings were photographed at 14 dpi (top) and 49 dpi (bottom) with mock or purified virions of CMV-2aTΔ2b (50 μg/mL). Bars, 1 cm. (**b**) Detection of CMV-2aTΔ2b CP in inoculated and systemic leaves of Col-0 and *esc1-1* at 7 dpi and 14 dpi by Western blotting, respectively. The bottom values represent the relative accumulation (RA) of CMV CP. The RbcL was stained by Coomassie blue as total protein loading controls. (**c**) Northern blotting analysis of CMV-2aTΔ2b RNA accumulation in inoculated leaves at 7 dpi and systemic leaves at 14 dpi. The bottom values represent the RA of the CMV RNA3. The band intensities were evaluated by ImageJ and the RA values of Col-0 were set as 1. Methylene blue-stained rRNA were used as loading controls for total RNAs.

To screen enhanced susceptibility to CMV (*esc*) mutants, we inoculated the M2 population of EMS-mutagenized Col-0 plants with CMV-2aTΔ2b. Consistent with our previous studies^12^, the 2b-deleted mutant CMV-2aTΔ2b did not cause visible symptom on wild-type Col-0 plants at either 14 days post inoculation (dpi) or 49 dpi due to RDR1- and RDR6-dependent RNA silencing amplification (Fig. 1a). In contrast, the *esc1-1* mutant developed severe systemic symptoms like curly leaves at 14 dpi and stunted growth at 49 dpi (Fig. 1a). Besides, the rosette leaves of *esc1-1* were slightly smaller than that of Col-0 and *esc1-1* plants were shorter than Col-0 (Fig. 1a). Western blotting and northern blotting analyses were further performed to assess virus accumulation in inoculated leaves and systemically infected leaves at 7- and 14- dpi, respectively. The *esc1-1* mutant plants accumulated similar levels of viral CP in inoculated leaves but higher level of viral CP in systemically infected leaves compared with those of wild type Col-0 plants (Fig. 1b). Likewise, northern blotting hybridization also showed that *esc1-1* mutant plants allowed an increased accumulation of viral genomic/subgenomic RNA in systemically infected leaves but maintained similar levels of viral RNAs in inoculated leaves compared to those of Col-0 plants (Fig. 1c).

To determine the relative accumulation of viral genome RNA3 and CP, the hybridization signal intensities were evaluated. The values of RNA3 and CP in virus-infected Col-0 plants were set as one unit. The analysis of systemically infected leaves revealed that the levels of CMV CP (Fig. 1b) and RNA3 (Fig. 1c) in the *esc1-1* mutant plants were 7 times more than those of Col-0 plants. While, there is no significant difference in the inoculated leaves of *esc1-1* mutant and Col-0 plants after infection of CMV-2aTΔ2b. Collectively, the systemic infection of CMV-2aTΔ2b was significantly enhanced in *esc1-1* mutant plants.

### Mapping and identification of *ESC1*

**Figure 2:**
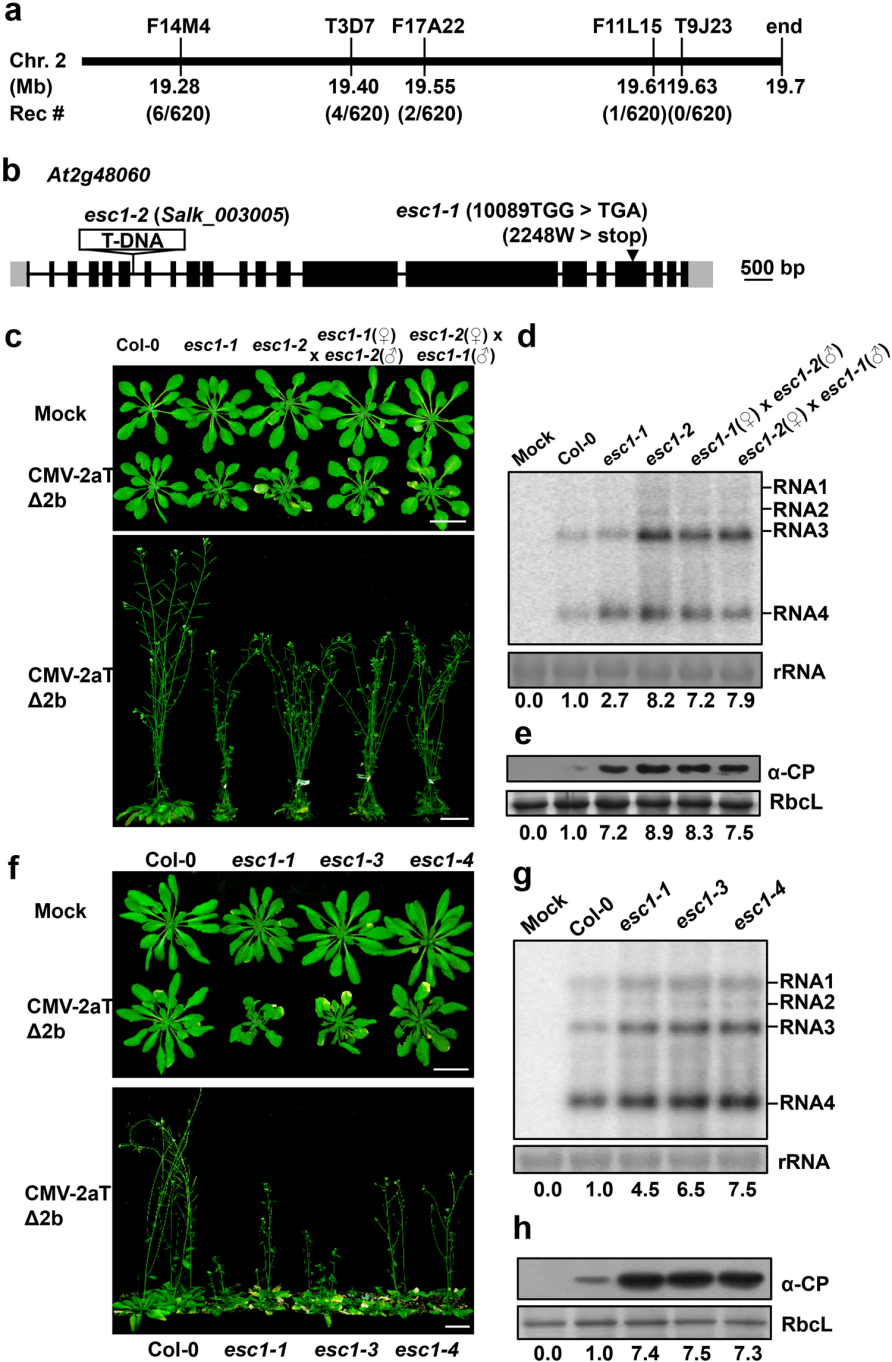
Mapping and cloning of *ESC1*. (**a**) Map-based cloning of *ESC1*. Five SSLP markers on chromosome 2 are indicated on the top. The physical distance of each markers and the numbers of informative recombinants from 310 F2 plants are shown below. Note that the mutation site was mapped to a 90-kb region at the end of chromosome 2. (**b**) Schematic diagram of *At2g48060* gene structure. Exons and untranslated regions of exons are indicated by black and gray boxes, respectively. The lines between the boxes represent introns. The mutation site of *esc1-1* in the 18th exon was indicated by an arrow head. Note that the T-DNA insertion of *esc1-2* (*Salk_003005*) is in the 6th intron. Bar, 500 bp. (**c**) Pathogenic responses of Col-0, *esc1-1*, *esc1-2*, and their F1 progeny at 14 days (upper) and 49 days (bottom) after infection with mock or CMV-2aTΔ2b. F1 plants were from the cross of *esc1-1* (female) and *esc1-2* (male) and a reciprocal cross as indicated. Bars, 3 cm. Accumulation of CMV-2aTΔ2b genomic/subgenomic RNAs (**d**) and viral CP (**e**) in the systemically infected leaves of the plant samples shown in (**c**) at 14 dpi. (**f**) Symptoms of Col-0, *esc1-1*, *esc1-3* and *esc1-4* inoculated with mock or CMV-2aTΔ2b at 14 dpi (upper) or 49 dpi (bottom). Bars, 3 cm. Accumulation of CMV-2aTΔ2b RNA (**g**) and viral CP (**h**) in the systemically infected leaves of the plant samples shown in (**f**) at 14 dpi. Methylene blue-stained rRNA were used as loading controls for total RNAs. In the **d** and **g**, the bottom values represent relative accumulation (RA) of the CMV RNA3. In the **e** and **h**, the bottom values represent RA of CMV CP. All the band intensities were evaluated by ImageJ and the RA values of Col-0 were set as 1. The RbcL was stained by Coomassie blue as protein loading controls.

In order to map *esc1-1* locus by a positional cloning strategy, the *esc1-1* mutant plants were crossed with wild-type Landsberg erecta (Ler) plants and the F2 population were developed. The resulting F2 population were further inoculated with CMV-2aTΔ2b and the segregation ratio was about 3:1 (resistant versus susceptible, Table 1). The F2 plants presented sensitive symptoms (Supplementary Fig. S1) were subjected to mapping-based cloning analysis with 34 simple sequence length polymorphisms (SSLPs). The low-resolution mapping showed that the mutation responsible for the *esc1-1* mutant was mapped to the position between 19.28 and 19.7 Mb of chromosome 2. Fine mapping using about 310 F2 plants narrowed down the mutation between 19.61 and 19.7 Mb (Fig. 2a).

**Table 1:** Genetic analysis of the sensitive phenotype in *A*. *thaliana* mutant *esc1*.

Plants	Resistant	Sensitive
Col-0	20	0
*esc1-1*	0	20
F2 (*esc1-1* × Col-0)	244^a^	88^a^
F2 (*esc1-1* × Ler)	1231^b^	381^b^
F1 (*esc1-1* × *esc1-2*)	0	20

The plants were inoculated with CMV-2aTΔ2b and resistance or sensitive phenotypes were exhibited at 14 dpi. aχ^2^(3:1) = 0.40, P > 0.05; bχ^2^(3:1) = 1.6, P > 0.05.

To further identify the point mutations in *esc1-1* mutant plants, the *esc1-1* mutant plants were backcrossed with Col-0 plants to generate F1 plants that further developed F2 population. Characterization of the disease susceptibility to CMV-2aTΔ2b showed that the ratio of resistant plants (244) to sensitive plants (88) was about 3:1 in the F2 population (Table 1), indicating that the *esc1-1* mutant is attributed to a single recessive gene mutation. Then, 50 F2 plants that displayed sensitive morphology (Supplementary Fig. S1) were collected to a bulked DNA pool for deep sequencing by Illumina. In the region between 19.61 and 19.7 Mb of chromosome 2, there is only a G to A point mutation leading to a premature stop codon at W2248 in the 18th exon of *At2g48060* (Fig. 2b and Supplementary Table S1).

To verify whether the premature stop mutation of *At2g48060* is responsible for the *esc1-1* phenotypes, a knockout mutant (*Salk_003005*, named *esc1-2*) with a T-DNA insertion in the sixth intron of *At2g48060* was obtained from Arabidopsis Biological Resource Center (ABRC) (Fig. 2b). The *esc1-2* mutant, like the *esc1-1* mutant, did not show abnormal developmental phenotypes (Fig. 2c). However, both *esc1-1* and *esc1-2* plants developed more severe disease symptoms after the CMV-2aTΔ2b infection compared with those of wild-type plants at 14- and 49- dpi (Fig. 2c). The F1 plants of a cross between *esc1-1* and *esc1-2* mutants exhibited similarly severe disease symptoms with those single mutants after the CMV-2aTΔ2b infection (Fig. 2c). Subsequently, northern blotting hybridization exhibited an increased virus accumulation in systemically infected leaves of the *esc1-1*, *esc1-2*, as well as their F1 progeny plants compared with Col-0 plants (Fig. 2d). Additionally, the single mutant of *esc1-1* and *esc1-2*, as well as their F1 plants allowed higher accumulations of viral CP than Col-0 plants (Fig. 2e). These results indicate that *esc1-1* and *esc1-2* are indeed allelic in the gene of *At2g48060*.

We further used the CRISPR/Cas9 system to generate *At2g48060* mutants through targeting its 19th exon (^10541^TATCAGCGCTTGCGAGGGGA^10560^, Supplementary Fig. S2). The resulting *esc1-3* and *esc1-4* contain a 5-bp deletion at 10555 bp and a 1-bp insertion at 10559 bp of *At2g48060*, respectively, both of which lead to frameshift mutations of *At2g48060* (Supplementary Fig. S2). Similar to *esc1-1* and *esc1-2* mutant plants, both *esc1-3* and *esc1-4* exhibited increased susceptible to CMV-2aTΔ2b infection and allowed enhanced accumulation of viral RNA and CP (Fig. 2f–h).

**Figure 3:**
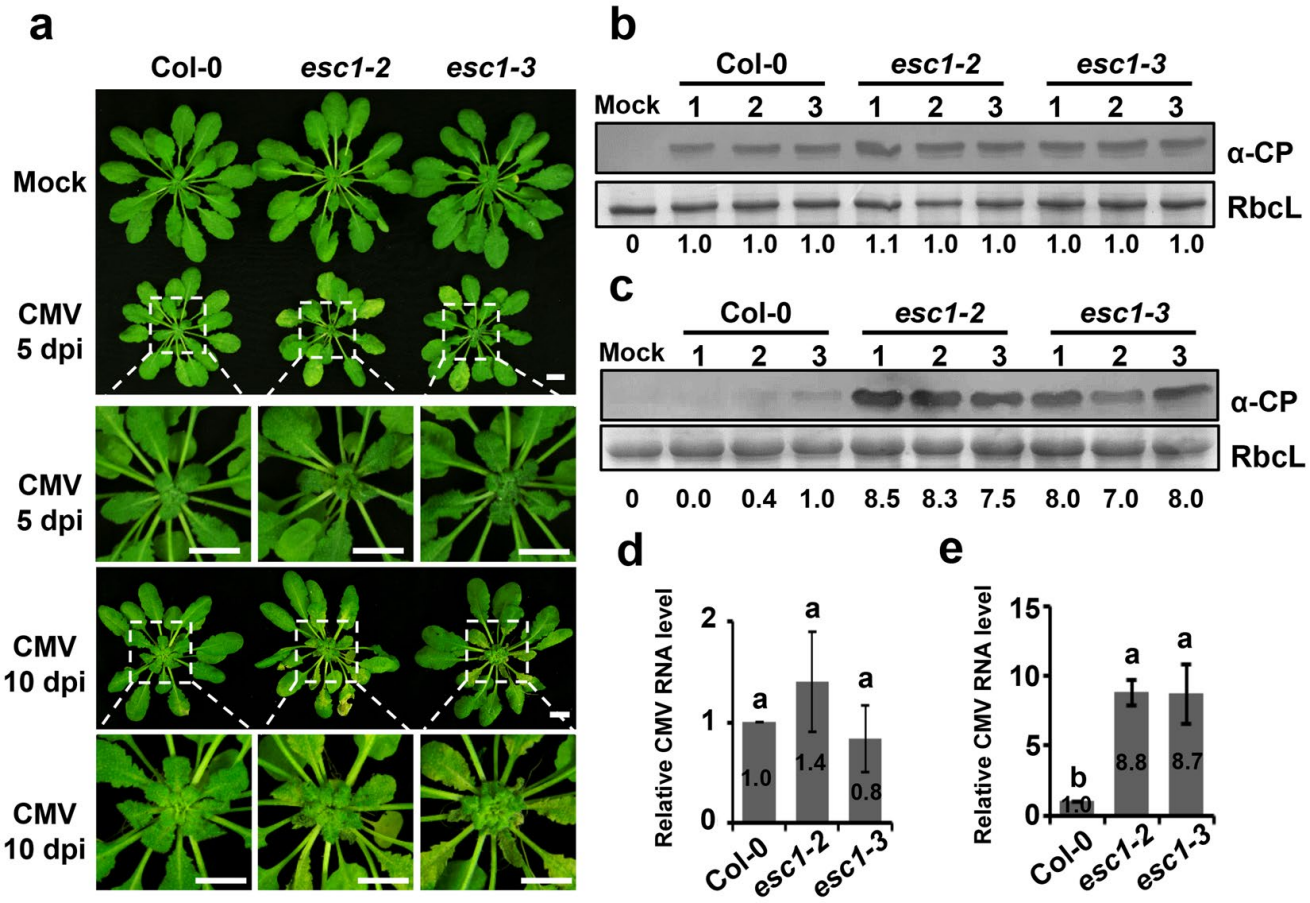
The *A*. *thaliana* mutant *esc1-2* and *esc1-3* exhibited enhanced susceptibility to wild type CMV. (**a**) Symptoms of *A*. *thaliana* Col-0, *esc1-2* and *esc1-3* inoculated with mock or wild type CMV at 5 dpi and 10 dpi. Col-0, *esc1-2* and *esc1-3* were inoculated with mock buffer or purified CMV virions (20 μg/mL). Bars, 1 cm. (**b**,**c**) Western blotting analysis to detect accumulations of CMV CP in inoculated leaves at 4 dpi (**b**) or systemically infected leaves at 5 dpi (**c**). Bottom values represent relative accumulations (RA) of CMV CP. The RbcL was stained as loading controls. Three independent repeats were indicated as 1, 2, and 3. (**d**,**e**) Detection of CMV RNA accumulation in inoculated leaves at 4 dpi (**d**) and systemic leavesat 5 dpi (**e**) by qRT-PCR. *Actin2* served as the internal control. The error bars represent SD from three independent experiments and letters above bars indicate statistically significant differences. According to the Duncan’s multiple range test, *P* > 0.05 in (**d**) and *P* < 0.01 in (**e**).

We further examined whether the *esc* mutants had enhanced susceptibility to wild-type CMV. To this end, Col-0 plants, *esc1-2*, and *esc1-3* mutants were challenged by CMV. As expected, the CMV-infected *esc1-2* and *esc1-3* mutants exhibited more severe symptoms than those of Col-0 (Fig. 3a). Western blotting analysis showed that similar accumulations of CMV CP were detected in inoculated leaves of Col-0 plants, *esc1-2*, and *esc1-3* mutants at 4 dpi (Fig. 3b), whereas systemically infected leaves of *esc1-2* and *esc1-3* mutants allowed significantly higher levels of CP than those of Col-0 plants (Fig. 3c). In consistence, quantitative real-time PCR analysis showed that CMV RNA accumulated to similar levels in all the inoculated leaves, but to a higher levels in systemically infected leaves of *esc1-2* and *esc1-3* mutants than those of Col-0 plants (Fig. 3d,e).

Taken together, all the *esc* mutants exhibited enhanced susceptibility to wild type CMV and the CMV-2aTΔ2b mutant. In addition, *At2g48060* is responsible for the ESC1-dependent viral immunity.

### ESC1 is a putative plant Piezo ortholog

**Figure 4:**
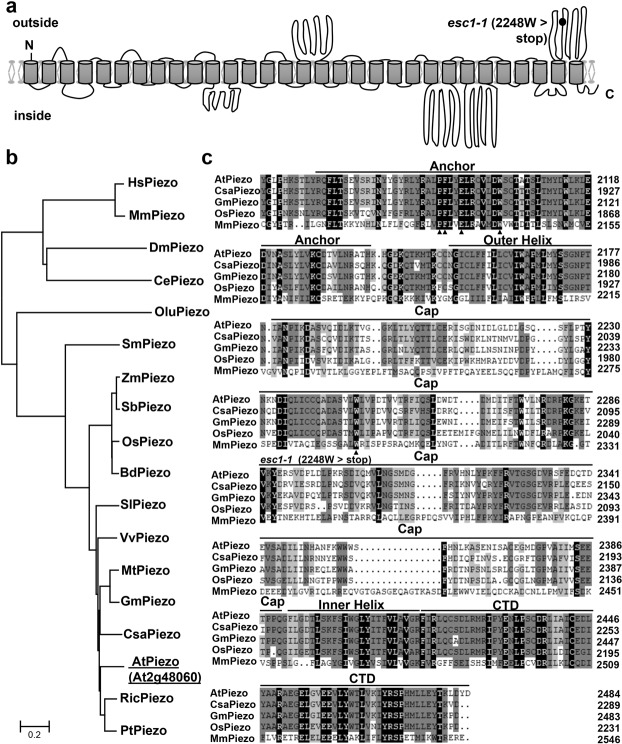
ESC1 is an ortholog of Piezo proteins. (**a**) Schematic diagram of predicted transmembrane topology of ESC1 (At2g48060). Transmembrane domains are indicated by grey boxes. The black dot indicates the premature stop codon in *esc1-1*. (**b**) Phylogenetic tree of ESC1/AtPiezo and 17 orthologs in animals and plants. ESC1/AtPiezo (At2g48060) is highlighted by underlining. *Homo sapiens* (Hs), *Mus musculus* (Mm), *Drosophila melanogaster* (Dm), *Caenorhabditis elegans* (Ce), *Ostreococcus lucimarinus* (Olu), *Selaginella moellendorffii* (Sm), *Zea mays* (Zm), *Sorghum bicolor* (Sb), *Oryza sativa* (Os), *Brachypodium distachyon* (Bd), *Solanum lycopersicum* (Sl), *Vitis vinifera* (Vv), *Medicago truncatula* (Mt), *Glycine max* (Gm), *Cucumis sativus* (Csa), *Ricinus communis* (Ric), *Populus trichocarpa* (Pt). (**c**) C-terminus alignments of AtPiezo and its orthologs of *C*. *sativus*, *G*. *max*, *O*. *sativa* and *M*. *musculus*. The highly conserved amino acids among Piezo orthologs and the mutation site of *esc1-1* are indicated by arrowheads. Anchor domain, Outer Helix, Cap domain, Inner helix and CTD domain are indicated on the above.

We cloned the cDNA of *At2g48060* that encoded a protein of 2485 amino acid (aa) residues (Supplementary Fig. S3). There is a 23 amino acid residues insertion behind the residue K403 compared with the predicted protein sequence of *At2g48060* from the available TAIR annotation (Supplementary Fig. S3, underlined and in bold). ESC1 contains 31 predicted transmembrane (TM) domains, and the point mutant of *esc1-1* mutant plants is located on the outside domain between the 30th and 31th transmembrane domain (Fig. 4a). Blast searching showed that ESC1 shares a high similarity with mammalian Piezo proteins (Fig. 4b). Many plant species, including monocot and dicot plants, have a single Piezo ortholog (Fig. 4b). Therefore, ESC1 is renamed AtPiezo, which is predicted in the previous reports^24,29,30^.

The cryo-electron microscopy structure of the mouse Piezo1 and chimer assays showed that its C-terminal region was responsible for ion-permeation properties^31–33^. Multiple alignments of the AtPiezo protein and its orthologs in *C*. *sativus*, *G*. *max*, *O*. *sativa* and *M*. *musculus* show that those C-terminal regions contain evolutionarily conserved motifs, including Anchor, Outer helix, Cap, Inner Helix, and CTD domains (Fig. 4c). Furthermore, amino acid residues P2129-F2130(X2)-E2133(X6)-W2140 of the mouse Piezo1 are also conserved in plant Piezo orthologs (Fig. 4c). The premature stop codon at W2248 in *esc1-1* plants results in deletion in the region Cap, Inner Helix, and CTD domains those are essential for ion-permeation properties^31–33^. Therefore, the AtPiezo of *esc1-1* plants is probably a loss-of-function mutation of ion channel pore properties.

### Tissue expression pattern of AtPiezo

**Figure 5:**
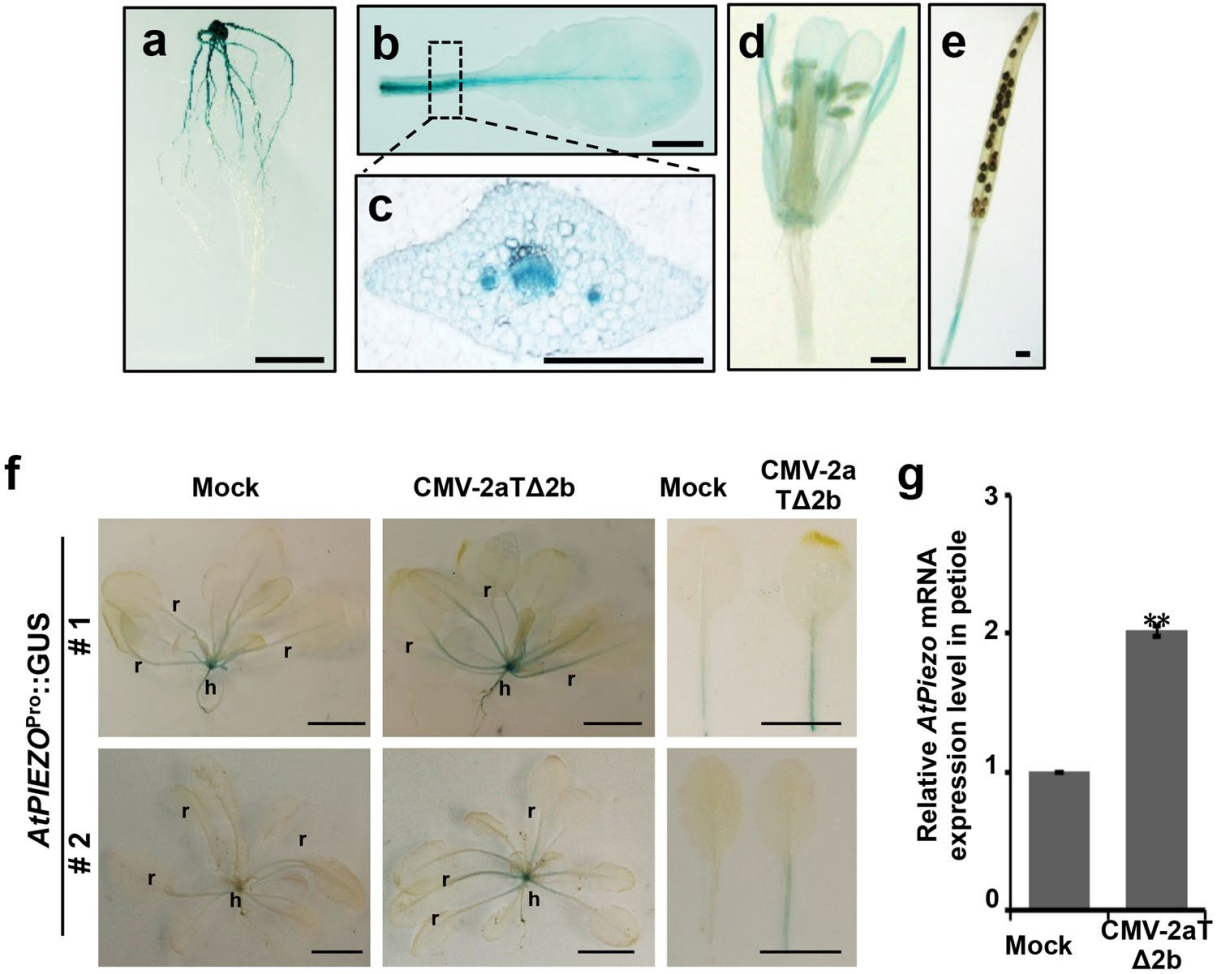
Tissue specific expression of AtPiezo. Expression analysis of *AtPiezo*^Pro^::GUS in different plant tissues. GUS activity was detected in root (**a**), petiole (**b**), flower petals (**d**) and silique stalk (**e**). Cross section of petioles (**c**) showed that GUS was highly expressed in the vasculature of petioles. Bars, 1 cm (**a**,**b**), 0.5 mm (**c**–**e**). (**f**) Two independent transgenic Col-0 plant lines harboring AtPiezo^Pro^-drived a GUS transgene were stained to show tissue specific expressions of GUS after mock treatment (left) and CMV-2aTΔ2b infection (middle) at 2 dpi. Detached leaves from the transgenic plants were shown in the right panels. Note the strong expression of GUS in the petioles. r, rosette leaves; h, hypocotyls. Bars, 1 cm. (**g**) Quantitative real-time PCR to detect the *AtPiezo* mRNA expression level in petioles of inoculated leaves by mock or CMV-2aTΔ2b at 2 dpi. Data points are the mean value of three independent experiments and error bars represent the standard deviation (s. d.). Asterisks indicate statistically signifcant difference according to Student’s *t*-test, **P-value < 0.01.

To investigate the spatial and temporal activities of the *AtPiezo* promoter, we analyzed β-glucuronidase (GUS) staining in tissues of transgenic Col-0 plants with the AtPiezo native promoter fused with a GUS reporter gene (*Piezo*^Pro^::GUS). GUS activity was detected in root, leaf petioles, flower petals and silique stalks, especially in vascular system of leaf petioles (Fig. 5a–e). In the mock (buffer)-treated plants, *Piezo*^Pro^::GUS was expressed mainly in the shoot meristem and the upper part of hypocotyl with a very weak level (Fig. 5f, left panel). In the CMV-2aTΔ2b-infected plants, *Piezo*^Pro^*::*GUS was expressed in a similar pattern but in a more expanded regions, especially in petioles of rosette leaves (Fig. 5f, right panel). In addition, the induction of *AtPiezo* in petioles of inoculated leaves by CMV-2aTΔ2b infection was revealed by quantitative real-time PCR (Fig. 5g). Collectively, our findings demonstrate that *AtPiezo* is transcriptionally induced by virus infection.

### AtPiezo conferred defense against systemic infection of turnip mosaic virus

**Figure 6:**
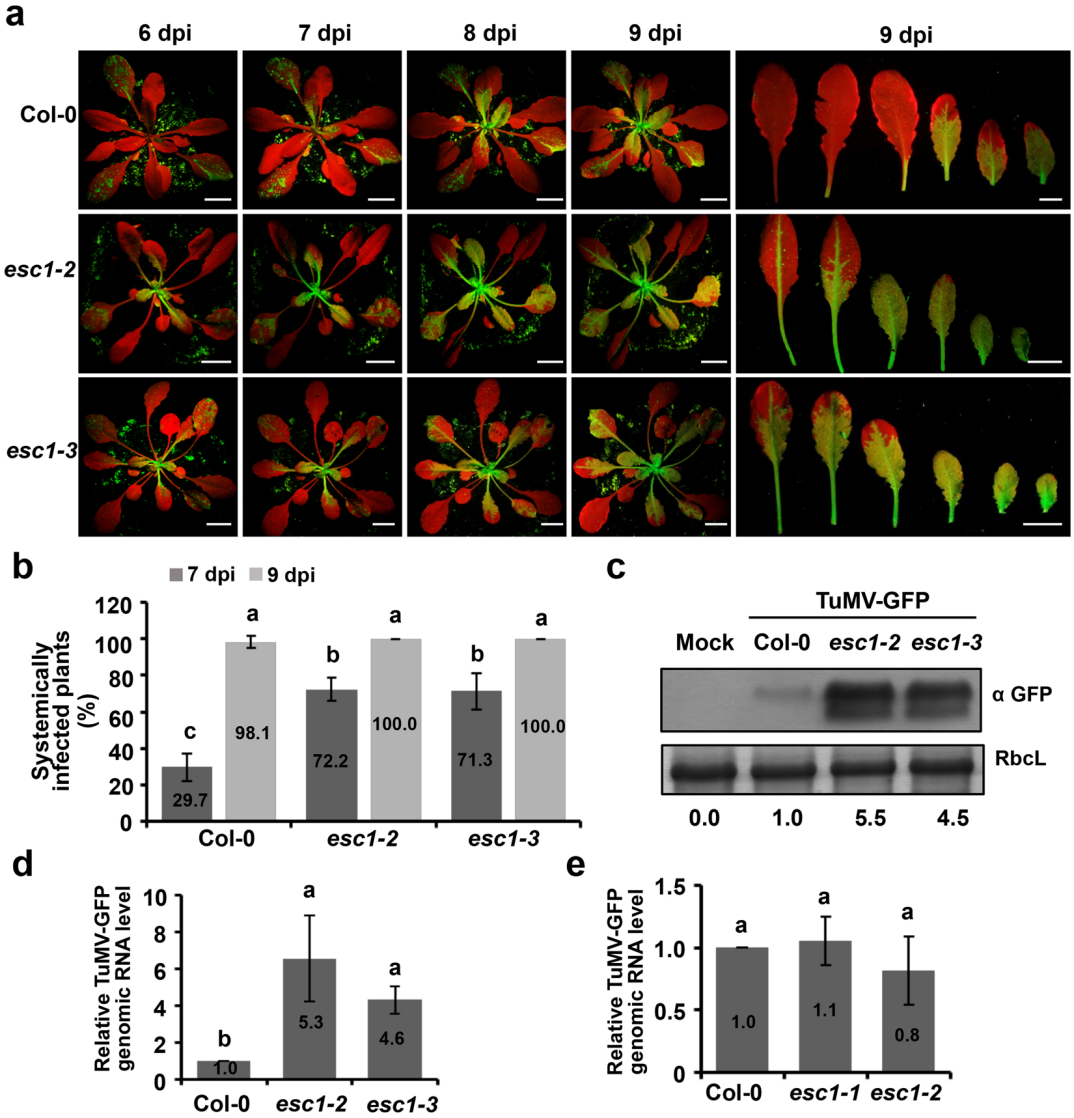
ESC1/AtPiezo mediates resistance to systemic infection of TuMV-GFP. (**a**) Photographs of Col-0, *esc1-2* and *esc1-3* inoculated with TuMV-GFP with a hand-held UV lamp at 6, 7, 8, and 9 dpi. Days post-inoculation are indicated above each panels. The representative images of systemically infected leaves at 9 dpi were shown on the right panels. Bars, 1 cm. (**b**) The percentage of systemically infected plants at 7 and 9 dpi. The percentage values are calculated according to total numbers of plants inoculated with TuMV-GFP. *P* < 0.01, n = 30. (**c**) Western blot analysis detecting GFP accumulations in systemically infected leaves of Col-0, *esc1-2* and *esc1-3* at 7 dpi. The bottom values represent the relative accumulation (RA) of GFP expressed from TuMV-GFP. All the band intensities were evaluated by ImageJ and the RA values of Col-0 were set as 1. (**d**) Quantitative real-time RT-PCR detecting accumulations of the TuMV-GFP genomic RNA in systemically infected leaves at 7 dpi. *P* < 0.05. (**e**) Accumulation of TuMV-GFP genomic RNA in protoplasts isolated from Col-0, *esc1-1* and *esc1-2* plants. Total RNA was extracted from protoplasts at 24 h post inoculation and subjected to qRT-PCR. *Actin2* served as the internal control. *P* > 0.05. In the (**b**,**d**,**e**), the error bars represent SD from three independent experiments. In each bar chart, letters above bars indicate statistically significant differences according to the Duncan’s multiple range test. *P* < 0.05 was considered significant differences and *P* < 0.01 was considered extremely significant differences.

To determine whether AtPiezo limits systemic infection of other plant viruses, wild-type Col-0 plants, *esc1-2* and *esc1-3* mutant plants were further inoculated by turnip mosaic virus (TuMV), a member of the *Potyviridae* family. To monitor the TuMV infection, we used an engineered strain with a GFP insertion (TuMV-GFP) and measured GFP fluorescence of infected plants at different time points after inoculation as described previously^19^. At 6 dpi, the infected *esc1-2* and *esc1-3* mutant plants exhibited obvious GFP fluorescence in the systemic full-expanded leaves, whereas only inoculated leaves rather than systemic leaves of Col-0 plants accumulated GFP fluorescence (Fig. 6a, left panels). Subsequently, the infected *esc1-2* and *esc1-3* mutant plants exhibited higher fluorescence intensity than wild-type plants at 7-, 8-, and 9- dpi (Fig. 6a). Of infected wild-type plants (n = 30), 29.7% were systemically infected by 7 dpi. By contrast, 72.2% of *esc1-2* and 71.3% of *esc1-3* mutant plants were systemically infected by TuMV-GFP by 7 dpi (Fig. 6b). By 9 dpi, almost all the infected plants exhibited GFP fluorescence in systemic leaves (Fig. 6b). However, the numbers of fully infected leaves of *esc1-2* and *esc1-3* mutant plants were greater than those of wild-type Col-0 plants at 9 dpi (Fig. 6a).

Western blotting was further performed to demonstrate that the GFP accumulation was increased in *esc1-2* and *esc1-3* mutant plants compared with Col-0 plants at 7 dpi (Fig. 6c). Moreover, quantitative real-time PCR analysis was conducted to examine accumulation of TuMV-GFP genomic RNAs in the upper systemically infected leaves, which showed that a significant increase of TuMV-GFP mRNA in *esc1-2* and *esc1-3* mutant plants than in wild-type Col-0 plants at 7 dpi (Fig. 6d). Similarly, *esc1-1* mutant plants also exhibited enhanced susceptibility to infection of TuMV-GFP (Supplementary Fig. S5). Meanwhile, TuMV-GFP RNA accumulations in the protoplasts isolated from *esc1-1* and *esc1-2* mutant plants were comparable to that in those of Col-0 plants (Fig. 6e). Collectively, these results suggested that AtPiezo plays a negative role in the systemic infection but not in the intracellular replication of TuMV-GFP.

The MCA protein family have MS channel activities to mediate mechanical signal-induced Ca^2+^ transport in regulation of cell growth and development^29,30^. To determine whether the MCA protein family have antiviral activities, the *mca1*, *mca2* single mutant plants, as well as *mac1 mac2* double mutant plants were confirmed to be null alleles and inoculated with TuMV-GFP (Supplementary Fig. S6). At 7 dpi, GFP fluorescence in the *mca1*, *mca2* single mutant plants, as well as *mca1 mca2* double mutant plants was comparable to wild-type Col-0 plants, while *esc1-2* plants exhibited enhanced susceptibility to infection of TuMV-GFP (Supplementary Fig. S6). These results indicate that MCA1 and MCA2, unlike AtPiezo protein, have no observable effects on systemic infections of TuMV-GFP.

## Discussion

Plants sense and respond to a wide variety of mechanical stimulation by mechanosensitive (MS) ion channels that provide pathways for ions movement from one side to the other side of the channels-targeted membranes. To date, three protein families have been proposed to be MS ion channels in *A*. *thaliana* including MSL, MCA, and Piezo families, although the molecular basis for these MS ion channels remain to be revealed. In contrast with MSL and MCA families, studies of which have gained breakthroughs in recent years^29,30,34,35^, plant Piezo proteins and their biological functions are largely unknown except their similarity with the well-studied animal Piezo orthologs^28,36^. Here, we took advantage of forward genetic screening to provide the first insight into the biological functions of plant Piezo orthologs in negatively regulating virus systemic spread (Figs 1 and 2). All the mutants of AtPiezo, including the premature stop mutant (*esc1-1*), the T-DNA inserted line (*esc1-2*), as well as two CRISPR/Cas9 mutants (*esc1-3* and *esc1-4*), exhibited enhanced susceptibility to CMV-2aTΔ2b infection in the systemic leaves but remained unchanged in inoculated leaves (Fig. 1b), indicating AtPiezo limits viral systemic infection (Figs 1, 3 and 6). These results demonstrate that the potential MS ion channel AtPiezo plays a role in restriction of systemic spread of a couple of viruses.

AtPiezo accumulated to very low level in mock plants (Fig. 5), which is probably responsible for the non-discernable developmental defect in mutant plants (Figs 1 and 2). Therefore, AtPiezo probably plays non-essential roles in plant development, which is the reason why the functions of plant Piezo have not been revealed in the previous studies. Nonetheless, *AtPiezo* is induced after virus infection suggesting that *AtPiezo* might be involved in virus immunity pathway. Besides, tissue-specific expression of virus-induced AtPiezo in the plant vasculature demonstrates that the negative effect of AtPiezo on viral systemic movement should occur in the vascular bundles. Indeed, induction of AtPiezo by virus infection in those petioles of rosette leaves probably impedes viral translocation and/or movement in phloem systems.

So far, only a few genes were identified in blocking virus long-distance movement. A cadmium-induced glycine-rich protein (cdiGRP) and a cdiGRP-interacting protein, named GrIP, restricts virus long-distance trafficking through improving callose deposition in phloem cells^3,4^. Genetic characterization of natural *Arabidopsis* accessions and mutants were employed to identify host genes involved in restricting tobacco etch virus movement (RTM). The results showed that at least five *RTM* genes were involved in defense against virus systemic infection^37–41^. In addition, the down-regulation of the subunit RPN9 of the 26S proteasome changes vascular development leading to inhibition of virus systemic infection^42^. Here, we used genetic analysis to show that AtPiezo suppresses systemic movement of plant viruses in *Arabidopsis thaliana*. Unfortunately, because two pairs of repeat sequences usually lead to a deletion from 1326 bp to 3315 bp of *AtPiezo* cDNA by intramolecular homologous recombination (Supplementary Fig. S4), we could not obtain the full-length cDNA of *AtPiezo* for biological and functional analysis.

## Methods

### Plant materials and growth conditions

Seeds of *A*. *thaliana* wild type in ecotype Columbia-0 (Col-0) and Lersberg erecta (Ler), as well as the T-DNA insertion mutant *esc1-2* (*Salk_003005*) were obtained from the Arabidopsis Biological Resource Center (ABRC, Columbus, OH). The *esc1-3* and *esc1-4* mutants were generated using a CRISPR/Cas9-based method as described previously^43^. Primers for the generation of CRISPR/Cas9 constructs are listed in Supplementary Table S2. *A*. *thaliana* seeds were sterilized by 10% bleach, plated on Murashige and Skoog (MS) medium supplemented with 3% sucrose and 1% agar, and vernalized at 4 °C for 3 days. Then, these plates were incubated in climate-controlled chambers at 22 °C for 7–10 days under a long day condition (16 h light/8 h dark). Seedlings were transferred into soil and grown in a growth room at 22 °C to 24 °C with a 10/14 h in light/dark program. To genotype the T-DNA insertion mutant, genomic DNA and RNA were extracted from the T-DNA insertion mutant leaves then PCR and RT-PCR were performed with gene-specific primers as listed in Supplementary Table S2.

### Virus inoculation

Four-week-old *A*. *thaliana* plants were mechanically inoculated with CMV-2aTΔ2b or CMV virions at the concentration of 50 μg/mL or 20 μg/mL as described previously^44^. The disease symptoms were recorded and virus CP and RNA were detected. *N*. *benthamiana* leaves infiltrated with TuMV-GFP were ground in 10 mM phosphate buffer with 2% PVP-40, pH 7.4, then the extract was used to mechanically inoculate 5-week-old *A*. *thaliana* plants as described previously^45^. The GFP fluorescence of plants inoculated with TuMV-GFP was photographed using a digital camera equipped with a yellow filter under a long wave length UV lamp (UVP, Upland, CA, USA) as described previously^46^.

### Forward genetic screen, map-based cloning and SNP analysis using next-generation sequencing

EMS mutagenesis in Col-0 background was performed as described previously^47^. Briefly, about 50,000 M1 seeds of Col-0 plants were incubated with 100 mM phosphate buffer (pH 7.5) 4 °C for 12 h, followed by mutagenized with 0.6% ethyl methanesulfonate (EMS) in phosphate buffer at 25 °C for 8 h. The M1 seeds were washed with sterilized water for 20 times and were grown in soil to generate the M2 population. The *esc1-1* was identified through screening susceptible mutants of the M2 populations after infection of CMV-2aTΔ2b. To identify the *ESC1* gene, *esc1-1* mutant plants were crossed with wild type Ler plants, and the resulting F1 plants were self-fertilized to generate F2 mapping populations. Genomic DNAs isolated from 310 susceptible F2 plants and 34 SSLP markers were used to narrow down the susceptible locus. Genomic DNA extracted from about 100 susceptible F2 plants generated from backcrossing *esc1-1* with Col-0 plants were utilized for SNP analysis by next-generation sequencing with Illumina HiSeq2000 platform. The wild type Col-0 genomic DNA was sequenced as a reference. Alignment of SNP analysis and reference DNA sequence was performed using SAM tools^48^. The SNP sites was visualized and confirmed by the Integrative Genome Viewer^49^.

### Phylogenetic analysis

To identify the coding region sequence of *AtPiezo* (*At2g48060*), three parts of the full CDS were amplified and inserted into pMD19T (Takara). The sequences were amplified with high fidelity DNA polymerase and all plasmids were verified by DNA sequencing. Primers used are listed in Supplementary Table S2. Protein sequences of other Piezo proteins were downloaded from the NCBI database (www.ncbi.nlm.nih.gov). Multiple alignment of amino acid sequences was performed with ClustalW^50^. The phylogenetic tree was generated using MEGA5 software implementing a neighbor joining method^51^. Phylogeny was indicated using the bootstrap method on 1000 replications.

### GUS staining

To clone the promoter of *AtPiezo*, the genomic region between 2.8 kb upstream and 45 bp downstream of the start codon of *AtPiezo* was introduced into the pBI101 vector (Clontech). The resulting plasmid containing *Piezo*^Pro^::GUS was transformed into Col-0 plants by the *Agrobacterium*-mediated floral-dipping method. *In vivo* GUS staining was carried out as described previously^14^. Two independent lines of *Piezo*^Pro^::GUS transgenic plants were examined for GUS expression at 2 days after treated by buffer or CMV-2aTΔ2b. Plant tissues were incubated with X‐Gluc solution (50 mM NaPO4 buffer, pH 7.0, 0.1% Triton X‐100, 10 mM potassium ferrocyanide, 10 mM potassium ferricyanide, and 3 mM 5‐bromo‐4‐chloro‐3‐indolyl‐β‐D‐glucuronide) at 37 °C for 14 h. Stained tissues were washed in an ethanol series.

### Quantitative real-time PCR

Total RNA was extracted from CMV or TuMV-GFP-infected systemic leaves of plants with TRIzol reagent (Invitrogen). After treated with RNase-free DNaseI (Takara), total RNA was used for the reverse transcription into cDNAs (MLV, Promega). To estimate the CMV CP mRNA and TuMV-GFP genomic RNA accumulation level, quantitative real-time PCR was performed with CMV CP and TuMV CP specific primers (Supplementary Table S2) using SsoFastTM EvaGreen® Supermix according to the manufacturer’s recommendations (Bio-Rad). The *A*. *thaliana Actin2* was used as an internal control. The primer pairs for qRT-PCR are listed in Supplementary Table S2.

### Protoplast analysis

*Arabidopsis* protoplasts were isolated from 4-week-old Col-0, *esc1-1* and *esc1-2* leaves as described previously^52^. About 10^6^ protoplasts of each sample were transfected with 50 μg plasmid of TuMV-GFP in 40% PEG4000 (Sigma) with 0.1 M CaCl_2_ and 0.8 M mannitol at 25 °C for 15 min. Transformation was stopped by W5 buffer (2 mM MES, pH 5.7, 154 mM NaCl, 125 mM CaCl_2_ and 5 mM KCl) and PEG4000 was removed by washing with W5 buffer, and then transformed protoplasts were incubated in W5 buffer in the dark for 24 h. Total RNA was extracted from protoplasts with TRIzol reagent (Invitrogen), and the viral RNA was analyzed by qRT-PCR.

### Data analysis

All experimental data from three independent repeat experiments were represented as mean ± SD. All statistical analyses were performed using one-way analysis of variance (ANOVA) by SPSS Statistics (SPSS Inc., Chicago, IL). *P* value < 0.05 was considered a significant difference and P value < 0.01 was considered an extremely significant difference.

### Accession numbers

Piezo sequence data from this study can be found in the GenBank data libraries under the following Accession Numbers: *Homo sapiens*, NP_001136336.2; *Mus musculus*, NP_001032375.1; *Drosophila melanogaster*, NP_001303314.1; *Caenorhabditis elegans*, CDR32751.1; *Ostreococcus lucimarinus*, XP_001418409.1; *Selaginella moellendorffii*, XP_002963641.1; *Zea mays*, XP_020405820.1; *Sorghum bicolor*, OQU86898.1; *Oryza sativa*, XP_015610648.1; *Brachypodium distachyon*, XP_010230896.1; *Solanum lycopersicum*, XP_010326620.1; *Vitis vinifera*, XP_010652101.1; *Medicago truncatula*, XP_013469247.1; *Glycine max*, XP_006588615.1; *Cucumis sativus*, XP_011659326.1; *Ricinus communis*, XP_015578213.1; *Populus trichocarpa*, XP_002321052.2.

## Supplementary information

Genetic analysis of a Piezo-like protein suppressing systemic movement of plant viruses in Arabidopsis thaliana supplymentary information

## Data Availability

The data has been submitted to the SRA database and the SRA Accession Number is SRP159419.
