# Supplementary material for: Genetic analysis of a Piezo-like protein suppressing systemic movement of plant viruses in Arabidopsis thaliana
Source: Sci Rep. 2019 Feb 28;9:3187. doi: 10.1038/s41598-019-39436-3 (PMC6395819; doi:10.1038/s41598-019-39436-3)
Supplement: Supplementary file 1 — Genetic analysis of a Piezo-like protein suppressing systemic movement of plant viruses in Arabidopsis thaliana supplymentary information [file 41598_2019_39436_MOESM1_ESM.docx]

**Genetic analysis of** **a Piezo-like protein** **suppressing systemic movement of plant viruses in *Arabidopsis thaliana***

Zhen Zhang^1^, Xin Tong^1^, Song-Yu Liu^1^, Long-Xiang Chai^1^, Fei-Fan Zhu^1^, Xiao-Peng Zhang^1^, Jing-Ze Zou^1^, Xian-Bing Wang^1*^

**Supplementary Information**

Supplementary Figures


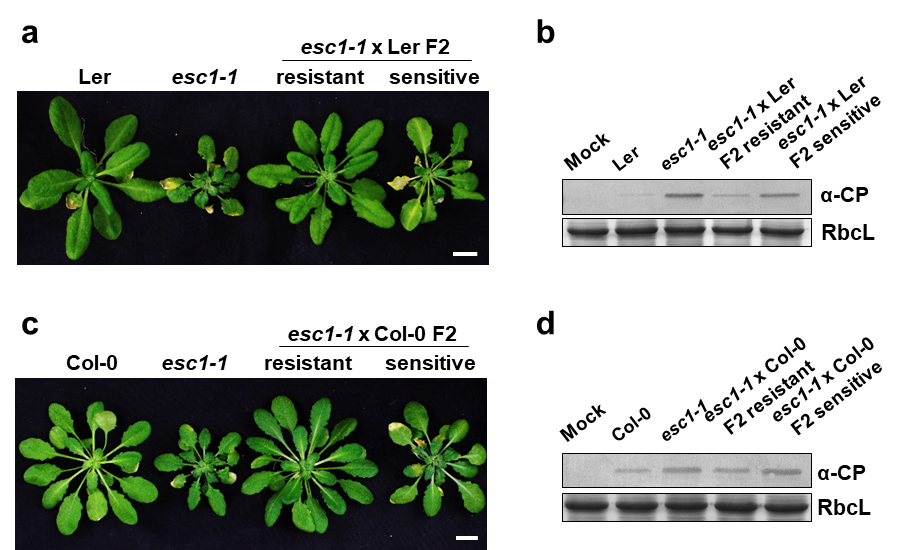


**Supplementary Figure S1. Pathogenic responses of the F2 progeny between *esc1-1* and Ler or Col-0 infected with CMV-2aTΔ2b.**

(**a**) The symptoms of Ler, *esc1-1* and their F2 progeny inoculated with CMV-2aTΔ2b at 14 dpi. Bar, 1 cm. (**b**) The CMV-2aTΔ2b CP in systemic leaves of Ler, *esc1-1* and their F2 progeny was detected by western at 14 dpi. (**c**) The phenotype of Col-0, *esc1-1* and their F2 progeny inoculated with CMV-2aTΔ2b at 14 dpi. Bar, 1 cm. (**d**) Detection of CMV-2aTΔ2b CP in systemic leaves of Col-0, *esc1-1* and their F2 progeny at 14 dpi. The RbcL was stained as loading controls.

**
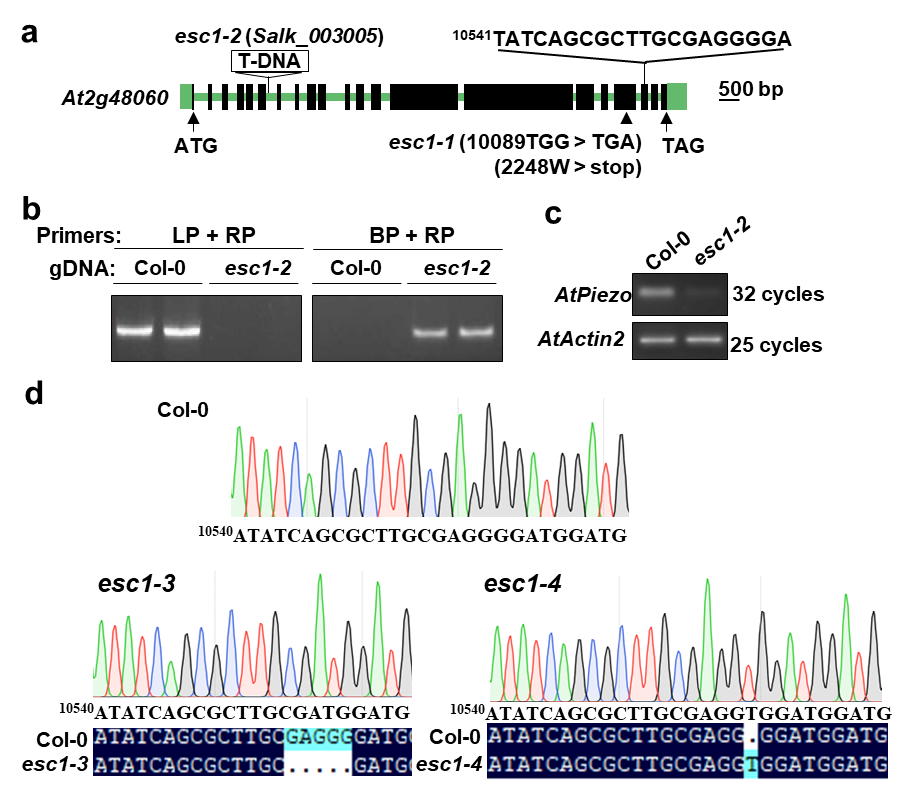
Supplementary Figure S2. Schematic diagram and genotyping of *esc1* mutants.**

(**a**) Schematic diagram illustrates mutation site of *esc1-1*, T-DNA insertion site of *esc1-2* (*Salk_003005*) and the selected target site for CRISPR/Cas9. The exons and UTRs are represented by black and gray boxes, respectively. Lines between the boxes represent introns. (**b**) Genomic DNA PCR demonstrates T-DNA insertion in *esc1-2* (*Salk_003005*) mutant. (**c**) RT-PCR shows nearly null expression of *ESC1*/*Piezo* transcript in *esc1-2* (*Salk_003005*) mutant. *Actin2* was amplified as a positive control. (**d**) Sequencing results of *esc1-3* and *esc1-4* mutants generated by CRISPR/Cas9 technology. Note that *esc1-3* and *esc1-4* harbor a 5-bp deletion at 10554 bp and a 1-bp insertion at 10559 bp of genomic DNA, respectively, both of which resulted in frameshift mutations.


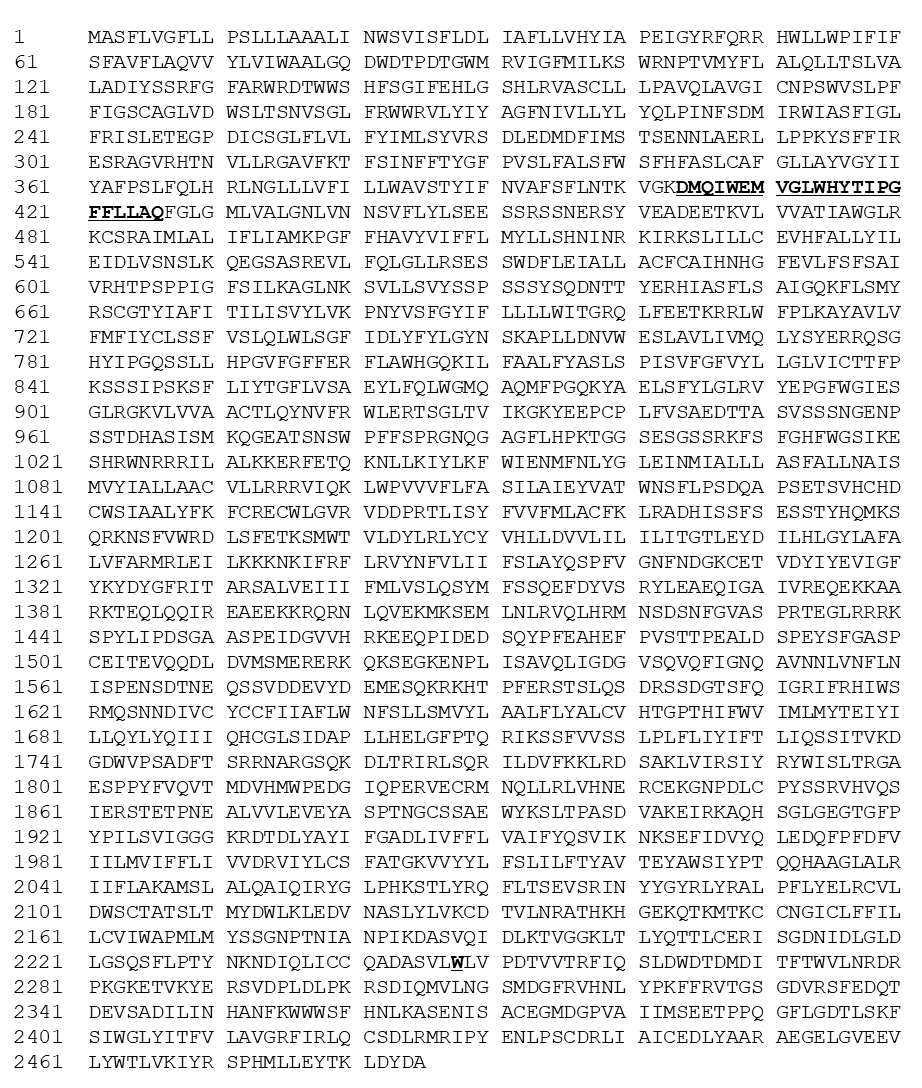
**Supplementary Figure S3. The protein sequence of ESC1/AtPiezo cloned from *A. thaliana.***

The coding sequence of *AtPiezo* was divided into 3 parts to be cloned into pMD19T-vector and sequenced. Note that AtPiezo cloned from *A. thaliana* contains a 23 amino acids insertion after 403K compared with the TAIR annotation. The 23 amino acids and mutation site of *esc1-1* mutant are labelled underlined and in bold.


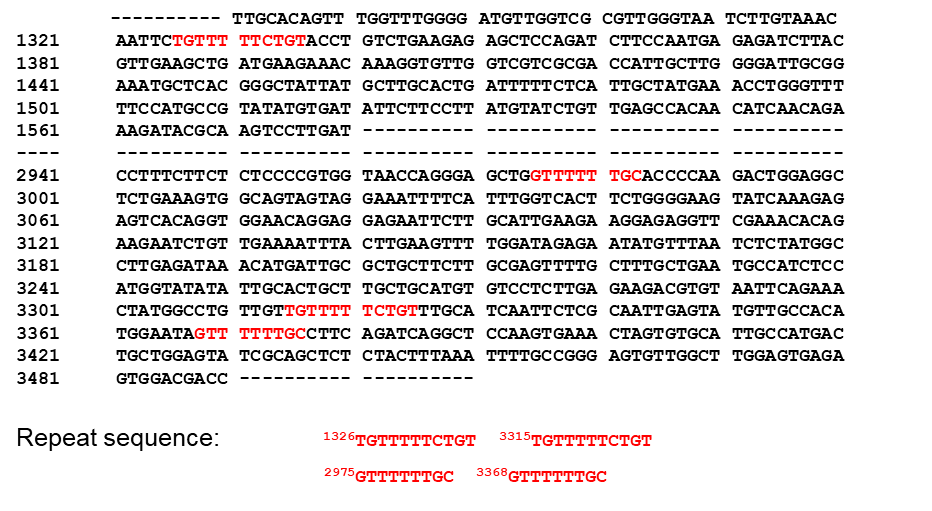


**Supplementary Figure S4.** **Two pairs of repeat sequences in the cDNA sequence of AtPiezo.**

The repeat sequences are labelled as red. Note that two pairs of repeat sequences induced intracellular recombination of *AtPiezo*.


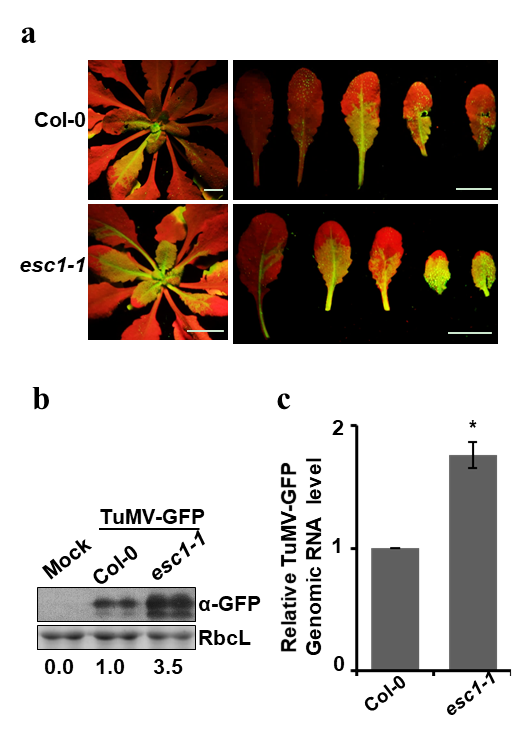


**Supplementary Figure S5. The *esc1-1* plants exhibited enhanced susceptibility to TuMV-GFP.**

(**a**) Symptoms of Col-0 and *esc1-1* inoculated with TuMV-GFP at 12 dpi. Representative images of systemic leaves were shown on the right panels. Bars, 1 cm. (**b**) Western blotting analysis detecting GFP accumulation in systemically infected leaves of Col-0 and *esc1-1* at 12 dpi. The RbcL was stained by Coomassie blue as protein loading controls. The bottom values represent the RA of GFP expressed from TuMV-GFP. All the band intensity was quantized by ImageJ and the RA values of Col-0 were set as 1. (**c**) Quantitative real-time RT-PCR to detect the TuMV-GFP genomic RNA accumulation in systemically infected leaves at 12 dpi. Data points are the mean value of three independent experiments. *P-value < 0.05.


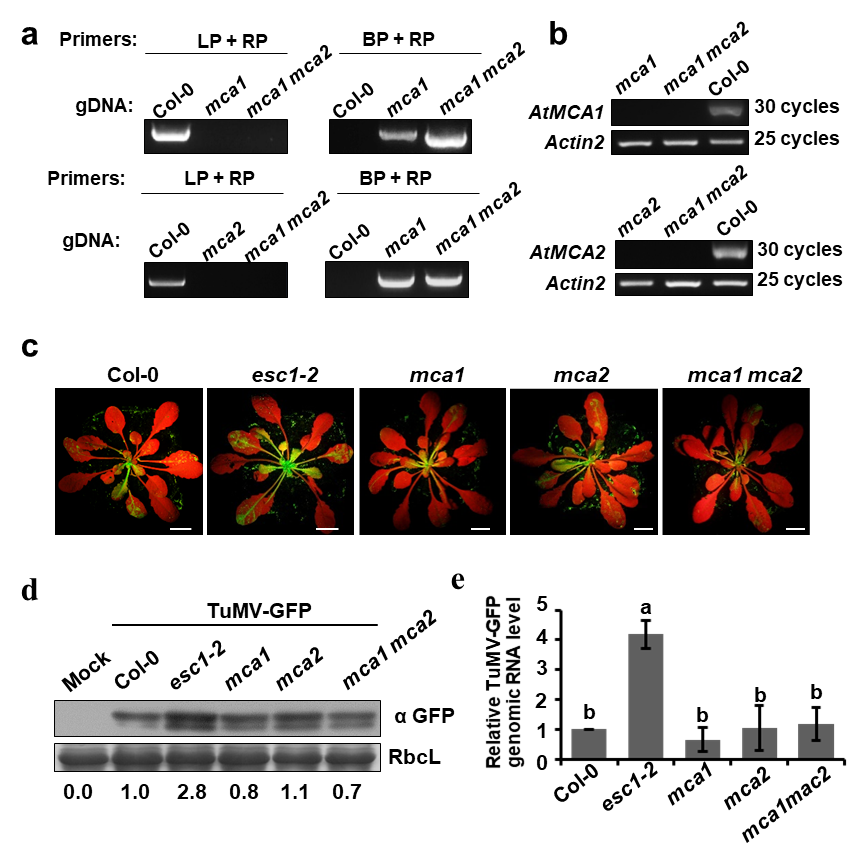


**Supplementary Figure S6. The antivirus examination of A. thaliana MCA genes.**

Genotyping of *mca1*, *mca2*, and *mca1 mca2* mutant plants by genomic DNA PCR (**a**) and RT-PCR (**b**) in *mca* mutants. (**c**) Photographs of Col-0, *esc1-2*, *mca1*, *mca2* and *mca1 mca2* inoculated with TuMV-GFP at 7 dpi. Photographs were taken with a hand-held UV lamp. (**d**) Western blotting analysis detecting GFP in systemically infected leaves of Col-0, *esc1-2*, *mca1*, *mca2* and *mca1mca2* at 7 dpi. The bottom values represent the RA of GFP expressed from TuMV-GFP. All the band intensity was quantized by ImageJ and the RA values of Col-0 were set as 1. The RbcL was stained by Coomassie blue as protein loading controls. (**e**) qRT-PCR detecting the TuMV-GFP genomic RNA accumulation in systemic leaves at 7 dpi. The error bars represent SD from three independent experiments, *P* < 0.05.

**Supplementary Table S1. The mutation site in *Arabidopsis* mutant *esc1-1* between 19.61 Mb and 19.70 Mb of chromosome 2.**

| **Chromosome** | **Location** | **Variation Type** | **Gene ID** | **Nucleotide change** | **Amino acid change** |
| --- | --- | --- | --- | --- | --- |
| Chr2 | 19652529 | Extron | *At2g48060* | TGG→TGA | W→* |

| **Supplementary Table S2. Primers in this study.** | |  |
| --- | --- | --- |
| **Name** | **Sequences (5'-3')** | **Comments** |
| pMD19T-AtPiezo-F1 | GCGACGCGTATGGCGAGTTTTTTGGTG | Construction of the *AtPiezo* gene |
| pMD19T-AtPiezo-R1 | GCCGGTACCAGTCGAAGATGGATTTTCACC | Construction of the *AtPiezo* gene |
| pMD19T-AtPiezo-F2 | GGTGAAAATCCATCTTCGACTGATCATGCTTCTATATCAATG | Construction of the *AtPiezo* gene |
| pMD19T-AtPiezo-R2 | GCGAATGCAAGATAACCCAGGTGTAGAATGTCATACTC | Construction of the *AtPiezo* gene |
| pMD19T-AtPiezo-F3 | TACCCGGGGATCCTCTAGACTGGGTTATCTTGCATTCGCTCTTGTTTTTG | Construction of the *AtPiezo* gene |
| pMD19T-AtPiezo-R3 | AGCCATGGAAGCTTGCATGCCTGCAGAGCATCATAGTCTAGCTTTGTATACTCG | Construction of the *AtPiezo* gene |
| LBb1.3 | ATTTTGCCGATTTCGGAAC | Detection of T-DNA, genotyping |
| SALK_003005 LP | CCCCTCAGTAAAACATTGGTG | Detection of T-DNA, genotyping |
| SALK_003005 RP | TCTTGTGGACTGGTCCTTGAC | Detection of T-DNA, genotyping |
| AtPiezo-F | AACAAGGCGAGGCAACGAGTAA | RT-PCR or qRT-PCR of *AtPiezo* mRNA |
| AtPiezo-R | TCCTCCTGTTCCACCTGTGACT | RT-PCR or qRT-PCR of *AtPiezo* mRNA |
| AtActin2-F | GCACCCTGTTCTTCTTACCG | RT-PCR or qRT-PCR of *AtActin2* mRNA |
| AtActin2-R | AACCCTCGTAGATTGGCACA | RT-PCR or qRT-PCR of *AtActin2* mRNA |
| DT1-BsF | ATATATGGTCTCGATTGTATCAGCGCTTGCGAGGGGAGTT | Construction of *esc1-3 and esc1-4* mutants by CRISPR/Cas9 system |
| DT1-F0 | TGTATCAGCGCTTGCGAGGGGAGTTTTAGAGCTAGAAATAGC | Construction of *esc1-3 and esc1-4* mutants by CRISPR/Cas9 system |
| DT1-R0 | AACACGTGAACCCTGCTAGAGTAAATCTCTTAGTCGACTCTAC | Construction of *piezo* mutant by CRISPR/Cas9 system |
| DT1-BsR | ATTATTGGTCTCGAAACACGTGAACCCTGCTAGAGTA | Construction of *piezo* mutant by CRISPR/Cas9 system |
| Cas9-target1F | GACCCTCTGGACCTTCCAAAACG | Detection of mutation in *esc1-3* and *esc1-4* mutants |
| Cas9-target1R | AGCATCATAGTCTAGCTTTGTATACTCGAG | Detection of mutation in *esc1-3* and *esc1-4* mutants |
| Cas9-target2F | TCTTCTGGGTCATCATGC | Detection of mutation in *esc1-3* and *esc1-4* mutants |
| Cas9-target2R | GTGACATTGCTTTGGCAAG | Detection of mutation in *esc1-3* and *esc1-4* mutants |
| AtPiezo-promoter-1 | GCCGGTACCGTTGGAAAGTGTCACACACGG | Construction of the promoter of the *AtPiezo* gene |
| AtPiezo-promoter-2 | ACGCGTCGACCAGAAGAAGCGAAGGCAACAAG | Construction of the promoter of the *AtPiezo* gene |
| GUS-F | AACTCAGCAAGCGCACTTACAG | PCR of *GUS* DNA orRT-PCR of *GUS* mRNA |
| GUS-R | GATTCACCACTTGCAAAGTCC | PCR of *GUS* DNA orRT-PCR of *GUS* mRNA |
| CMV-CP-F | CCTTTGTAGGGAGTGAACG | qRT-PCR of CMV CP mRNA |
| CMV-CP-R | CCCACACGGTAGAATCAA | qRT-PCR of CMV CP mRNA |
| TuMV-CP-F | GTGATGATGGACGGTGATGA | qRT-PCR of TuMV-GFP CP mRNA |
| TuMV-CP-R | TGTATGGTCGGTCTTGGTTAC | qRT-PCR of TuMV-GFP CP mRNA |
| **Name** | **Sequences (5'-3')** | **Comments** |
| 2-AC004411-9789-F | AGTTCCGAGGGGCGAGGG | SSLP marker F14M4 |
| 2-AC004411-9789-R | GTATGCACCGCAACAATC | SSLP marker F14M4 |
| T3D7-F | GGTATCGATTGAGCAAATAA | SSLP marker T3D7 |
| T3D7-R | ACATGCGTCTGCTTGGAG | SSLP marker T3D7 |
| 2-AC005309-9927-F | ACGAATATTGATTGTCTAAG | SSLP marker F17A22 |
| 2-AC005309-9927-R | AACCTAAGGGAAGGCTAC | SSLP marker F17A22 |
| 2-AC006072-9954-F | AAACTATGCATGGTATTAGC | SSLP marker F11L15 |
| 2-AC006072-9954-R | CCTCGATAACTATTACTCC | SSLP marker F11L15 |
| 2-AC006072-9963-F | TCGGTCTCTCTGGTCTTC | SSLP marker T9J23 |
| 2-AC006072-9963-R | ATCGCTCTGCGCAACCAA | SSLP marker T9J23 |
